# High-gamma tACS may regulate brain network connectivity to alleviate symptoms in female adolescent non-suicidal self-injury: a preliminary TMS-EEG pilot study

**DOI:** 10.1186/s12888-026-07978-2

**Published:** 2026-03-19

**Authors:** Wensi Hao, Xuemeng Ding, Kenan Ren, Chang Cui, Yuqing Zhang, Jiahui He, Xin Guo, Yuping Wang, Haiyan Ju, Yicong Lin

**Affiliations:** 1 Department of Neurology, Xuanwu Hospital, Capital Medical University, No. 45 Changchun Street, Beijing, 100053 China; 2 Beijing Key Laboratory of Neuromodulation, Beijing, China; 3 Neuropsychiatric Treatment Center, Hebei Hospital of Xuanwu Hospital, Shijiazhuang, China

**Keywords:** Non-suicidal self-injury, Transcranial alternating current stimulation, TMS-EEG, Brain network, Functional connectivity

## Abstract

**Objectives:**

To explore the efficacy and potential neurophysiological mechanisms of 77.5 Hz transcranial alternating current stimulation (tACS) in the treatment of female adolescent non-suicidal self-injury (NSSI).

**Methods:**

Six female NSSI patients received 21 days of 77.5 Hz, 15 mA tACS treatments. Neuropsychological scales were assessed at baseline (W0), after treatment (W3), and 4-week (W7) and 8-week (W11) follow-ups. Transcranial magnetic stimulation with EEG evaluated changes in source-level brain activity and phase-synchronous functional connectivity. Mixed repeated-measures ANOVA with Bonferroni correction (*p* < 0.017) was used for behavioral data analysis to correct for multiple comparisons. Effect sizes (Cohen’s d) was reported for all statistical results.

**Results:**

Significant improvements were observed in depressive symptoms and self-injury behaviors after treatment (OSIC: W7: *P* = 0.009, Cohen’s d=-1.682; HAMD-24: W3: *P* = 0.006, Cohen’s d=-1.892; W7: *P* = 0.001, Cohen’s d=-2.839; W11: *P* = 0.001, Cohen’s d=-2.738; all *P* < 0.017). Electrophysiological analysis revealed that 77.5 Hz tACS might decrease Default network activity, increased Limbic, SalVAttn and Control network activity, and enhanced the functional connectivity in high-gamma band between Control and SalVAttn/Default network. A positive correlation was found between increased C100 activity in the SalVAttn (left frontal-insula) region and reduced HAMD-24 scores (*R* = 0.826, *p* = 0.043), this correlation analysis was based on a small sample size (*n* = 6), and the correlation coefficient was unstable, with results only for preliminary exploratory reference.

**Conclusion:**

77.5 Hz tACS may alleviate NSSI symptoms in female adolescents potentially by regulating brain activity and functional connectivity in emotional-control networks.

**Supplementary Information:**

The online version contains supplementary material available at 10.1186/s12888-026-07978-2.

## Highlights

Our study provides the first preliminary evidence that 77.5 Hz tACS significantly may reduce self-injury behaviors and depressive-anxiety symptoms in female adolescent depressive disorder with NSSI.

77.5 Hz tACS may ameliorate NSSI symptoms in female adolescents by attenuating hyperactivity in the Default network region associated with rumination, and increasing activity in the Limbic, SalVAttn and Control network regions.

77.5 Hz tACS may enhance high-gamma top-down emotional-control network connectivity between Control and SalVAttn/Default networks regions in female adolescents with NSSI.

**Supplementary Information:**

The online version contains supplementary material available at 10.1186/s12888-026-07978-2.

## Introduction

Adolescent depressive disorder, marked by a high incidence and significant societal impact, often co-occurs with non-suicidal self-injury (NSSI) [1]. NSSI is defined as deliberate, repetitive self-injury without suicidal intent, though it is closely associated with suicidal tendencies [2]. The global prevalence of NSSI among adolescents is 17%, rising to 45% in China [3]. Current psychological and pharmacological treatments show limited efficacy, contributing to low remission rates [3].

Recent advances in neuroimaging have highlighted neuromodulation as a promising therapeutic option for NSSI. Studies suggest that NSSI is associated with abnormalities in intra- and inter-network connectivity within the emotional-control network (Limbic, Salience Ventral Attention(SalVAttn), Control, and Default) [4, 5], which may underlie impaired emotional perception, interoceptive awareness, rumination and emotional regulation [6]. The prefrontal cortex (PFC), particularly the dorsolateral prefrontal cortex (DLPFC) and orbitofrontal cortex (OFC) within the Fp1/Fpz/Fp2 region, is a core component of the emotional-control network [4, 5]. Previous studies have demonstrated that PFC hypoactivation and disrupted connectivity are key neurophysiological features of NSSI and depression [5, 7], and targeted neuromodulation of the PFC can modulate emotional regulation function [8, 9]. Quantitative electroencephalogram (EEG) analyses have shown reduced gamma oscillation (30–100 Hz) power and phase synchronization in Control (FP1, F3, F7) and other networks, correlating with depression severity [7, 10]. These findings suggest an excitation-inhibition imbalance and disrupted connectivity within and between emotional-control network in NSSI patients, particularly in the high gamma frequency range [11].

Transcranial alternating current stimulation (tACS) induces endogenous neural oscillations by applying exogenous sinusoidal current, thereby enhancing synaptic plasticity and phase synchronization across networks [12]. Studies have shown that 77.5 Hz tACS at 15 mA (peak-to-peak amplitude) has been established as an effective treatment for major depressive disorder [8, 9, 13], enhancing synchronization within and between brain networks through long-term potentiation [14]. Therefore, tACS holds potential as a therapeutic intervention for NSSI by modulating the disrupted connectivity within the emotional-control network.

Transcranial magnetic stimulation combined with EEG (TMS-EEG) allows for trans-synaptic activation of neural networks with millisecond temporal resolution [15], reflecting the inhibitory-excitatory balance and functional connectivity within neural circuits, thereby providing neurophysiological validation.

We hypothesize that 77.5 Hz tACS may alleviate NSSI symptoms in adolescents by regulating the inhibitive-excitatory balance, improving intra-network functional integration and long-range connectivity across neural networks measured by TMS-EEG.

## Methods

We recruited six right-handed patients (6 female) diagnosed with adolescent depressive disorder with non-suicidal self-injury behavior at the Department of Neurology of the Hebei Hospital of Xuanwu Hospital, Capital Medical University between December 2024 and January 2025. This study was conducted in accordance with the Declaration of Helsinki and relevant ethical guidelines, with ethics approval [Approval No. LYS (2024) 164] in October 2024 and informed consent obtained from the legal guardians of all patients. This pilot experiment serves as a preliminary exploration for a larger randomized controlled trial (RCT), which has been registered in the Chinese Clinical Trial Registry (ChiCTR) under the identifier ChiCTR2600118169.

**Fig. 1:**
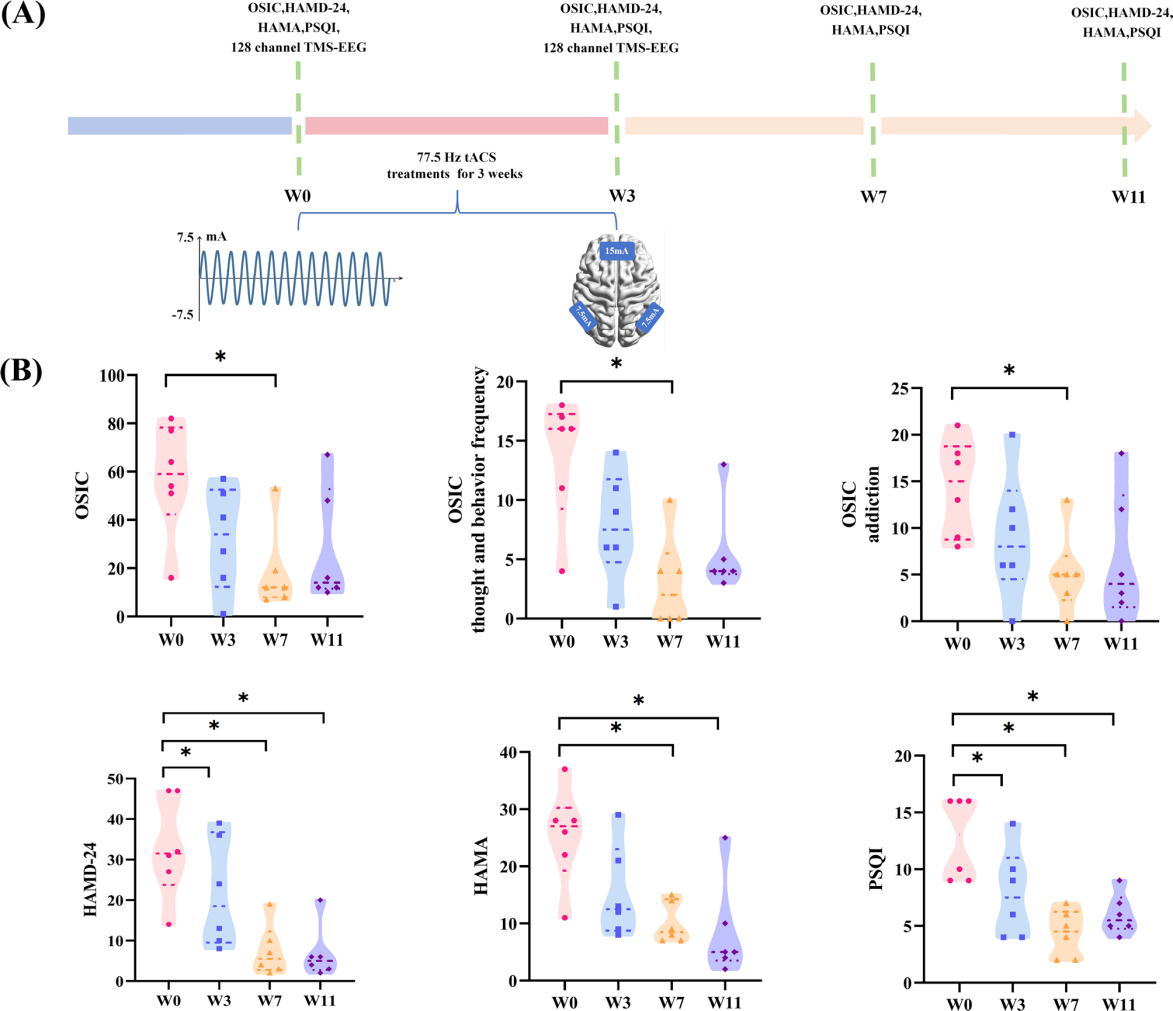
Trial flowchart and clinical outcome. (**A**) Overview of the study design. (**B**) Scores on psychometric scales at baseline (W0), after treatment (W3), and at 4-week (W7) and 8-week (W11) follow-ups in female NSSI patients. NSSI: Non-suicidal self-injury; OSIC: Ottawa Self-Injury Inventory Chinese Revised Edition; HAMD-24: Hamilton Depression Scale-24; HAMA: Hamilton Anxiety Scale; PSQI: Pittsburgh Sleep Quality Index. ^*^P < 0.017 vs. W0 (after bonferroni correction)

The patient evaluation and treatment protocol were illustrated in Fig. 1A. Patients received 21 consecutive days of 77.5 Hz, 15 mA (peak-to-peak amplitude) tACS (Nexalin Technology, Inc) treatment [9] with 42 sessions (two 40-minute sessions per day with ≥ 2-hour intervals). Real-time impedance monitoring was performed during all tACS sessions, and stimulation was automatically suspended by the device when impedance exceeded 5 kΩ to avoid skin irritation, patients’ vital signs and subjective discomfort were closely monitored during stimulation. A follow-up record of adverse events (AEs) was conducted throughout the treatment and follow-up period. A 4.45 × 9.53 cm electrode were placed on the prefrontal cortex (Fp1, Fpz, Fp2) and two 3.18 × 3.81 cm electrodes were placed on the bilateral mastoids, with the current density calculated as follows: mastoid region 6.19 A/m² (15 mA/(3.18 cm×3.81 cm×2)), prefrontal region 3.54 A/m² (15 mA/(4.45 cm×9.53 cm)), both lower than the 6.3 A/m² safety limit proposed by Bikson et al. [9, 16]. Neuropsychological assessments, including the Ottawa Self-Injury Inventory Chinese Revised Edition (OSIC), Hamilton Depression Scale-24 (HAMD-24), Hamilton Anxiety Scale (HAMA), and Pittsburgh Sleep Quality Index (PSQI) [11, 12], were conducted at baseline (W0), after treatment (W3), and 4-week (W7) and 8-week (W11) follow-ups. Clinical efficacy was analyzed using mixed repeated-measures ANOVA with bonferroni correction (*p* < 0.017). Effect sizes (Cohen’s d) was calculated for all behavioral scale results. We explicitly predefined “clinical response” prior to data analysis, consistent with standard criteria for tACS and depression/self-injury intervention studies [3, 9]: For depressive symptoms (HAMD-24): A ≥ 50% reduction in total score from W0 to W7, or a final score ≤ 7 points (indicating minimal to no depressive symptoms). For self-injury behaviors (OSIC): A ≥ 40% reduction in total score from W0 to W7 (adapted for adolescent NSSI, as self-injury behaviors are often more persistent than depressive symptoms [3]).

128-channel TMS-EEG targeting the F3 site was recorded before and after treatment to analyze TMS-evoked potentials (TEPs) and source-level brain activity. We preprocessed EEG data before and after treatments in patients with NSSI using MATLAB (R2023a), EEGLAB software (The MathWorks Inc., Natick, MA, 2022) and TESA [17] (An open source extension of EEGLAB). First, TMS-EEG data were introduced. At least 100 data segments were retained for each patient, and EEG data were intercepted before 1000 ms to 2000 ms after single-pulse TMS. The filter bandwidth was adjusted to 1–90 Hz, and we performed notch filter removal on the data (48–52 Hz). Independent component analysis (ICA) was used to remove artifacts, including eye movement (EOG), muscle (EMG) and TMS-induced artifacts (TMS pulse artifacts were removed by TESA’s built-in artifact subtraction algorithm) [18]. The data for each subject was superimposed to average to obtain the TEPs with the average amplitude of the 500 ms before stimulation as baseline correction.

Brainstorm was used to coregister the reconstructed the default headmodel surface and TMS-EEG data. A standardized low resolution brain electromagnetic tomography (sLORETA) was used to trace the source of TMS-EEG signals. Source localization was based on Brainstorm’s default MNI head model with a spatial resolution of 3 mm × 3 mm × 3 mm; inter-individual anatomical variability (e.g., differences in brain structure and skull thickness) may lead to a small deviation (about 5–10 mm) in the source localization results, and the reported brain region activations are regional positional references rather than precise anatomical localizations. After artifact rejection, the residual artifact amplitude was less than 2 standard deviations of the baseline signal, the signal-to-noise ratio (SNR) of the high-gamma band signal was greater than 3 dB in all subjects, and the consistency of source localization results before and after artifact removal was greater than 85%.

High-gamma band (60–90 Hz) data extraction: time-frequency analysis was performed using Morlet wavelet transform, and C100 (70–150 ms) and C200 (150–260 ms) time window data were extracted for subsequent connectivity analysis. Finally, the estimated source activities were assigned to 100 cortical regions of interest (ROIs) based on the scaefer100-Yeo7 atlas for further analysis. The 100 ROIs can be assigned into seven distinct brain networks: (1) visual network, (2) somatomotor network (SomMot), (3) dorsal attention network (DorsAttn), (4) salience/ventral attention network (SalVAttn), (5) limbic network, (6) control network, (7) default mode network (Fig S1).

Given that the weighting approach of the weighted phase lag index (wPLI) improved the signal - to - noise ratio, making it more suitable for analyzing TMS - induced connectivity changes, wPLI [19] was employed to quantify high - gamma (60–90 Hz) phase synchronization, and paired t - tests were used to assess TEPs and functional connectivity (*p* < 0.05). Pearson correlation coefficient were calculated to examine relationships between source-level activity changes and clinical improvements (see supplementary eMethods).

## Results

The study included 6 female NSSI patients (mean age = 14.17 ± 0.70 years, course of disease = 18.83±2.92  months, mean education = 7.33 ± 0.61 years), all of whom completed the 8-week follow-up without significant adverse effects. During the treatment period, only one patient experienced mild headache, but the symptoms were completely recovered within a short period of time. No other adverse events were observed during the entire treatment and follow-up period, and all patients completed the treatment with good tolerance. Neuropsychological assessments showed time-dependent improvements (Fig. 1B, Table S1). Significant reductions in depressive symptoms and self-injury behaviors were observed after treatment compared with baseline (OSIC: W7: *P* = 0.009, Cohen’s d=-1.682; HAMD-24: W3: *P* = 0.006, Cohen’s d=-1.892; W7: *P* = 0.001, Cohen’s d=-2.839; W11: *P* = 0.001, Cohen’s d=-2.738; all *P* < 0.017), with sustained decreases in self-injury thoughts, and addiction at 4-week follow-up (W7: *P* = 0.004; Cohen’s d=-2.114; *P* = 0.007, Cohen’s d=-1.820; both *P* < 0.017). Anxiety and sleep quality also improved significantly, with reductions in HAMA and PSQI scores lasting through follow-up (HAMA: W7: *P* = 0.004, Cohen’s d=-2.081; W11: *P* = 0.002, Cohen’s d=-2.467; PSQI: W3: *P* = 0.014, Cohen’s d=-1.516; W7: *P* = 0.000, Cohen’s d=-3.585; W11: *P* = 0.005, Cohen’s d=-1.969; all *P* < 0.017).All patients met the clinical response criterion for HAMD-24. 5 out of 6 patients (83.3%) met the clinical response criterion for OSIC.

**Fig. 2:**
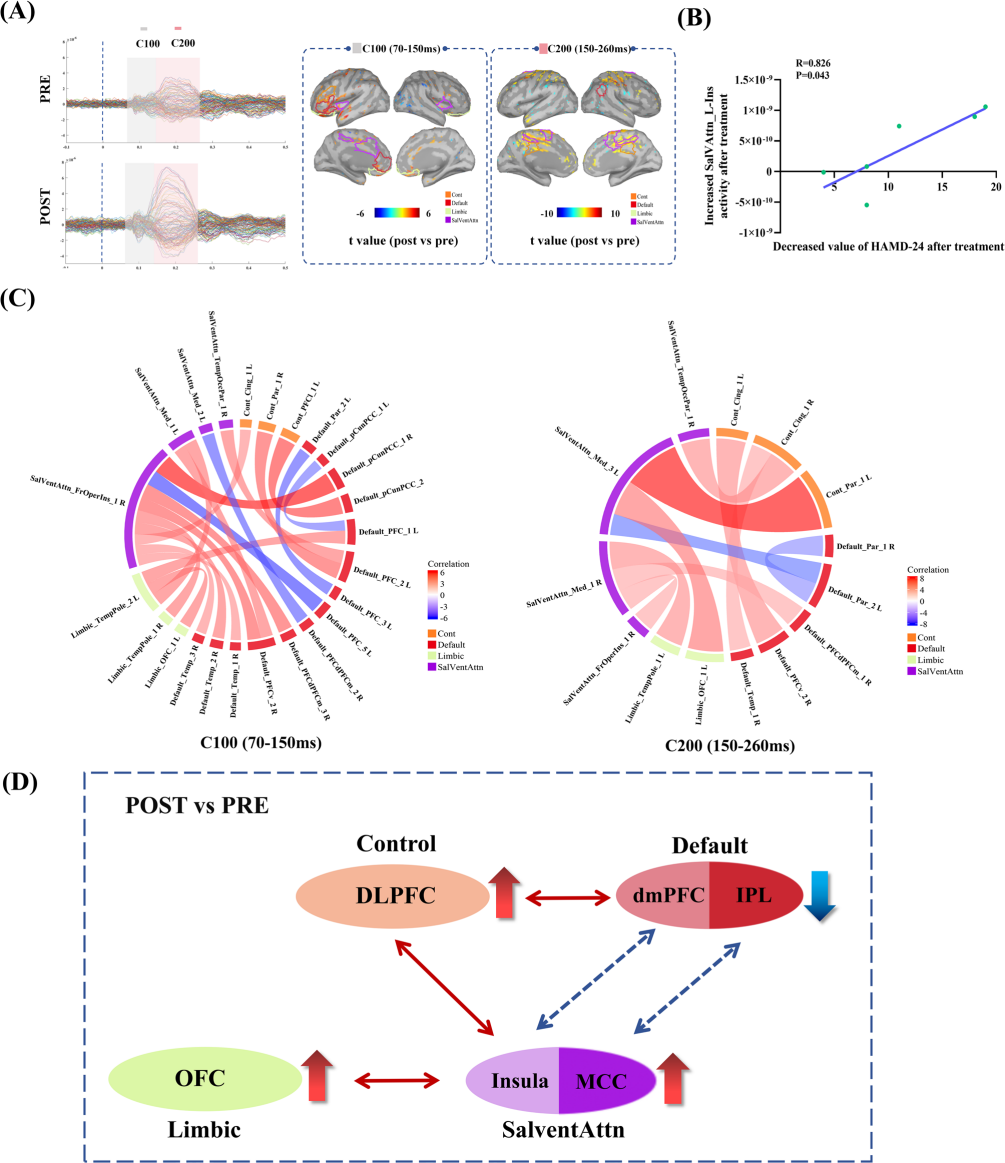
Changes in TMS-evoked potentials (TEPs) at source estimated level and weighted phase lag index (wPLI) phase synchronous in female NSSI patients before and after treatment. (**A**): Changes in average TEPs and estimated source-level activity evoked from left dorsolateral prefrontal cortex (F3 electrode) in female NSSI patients before and after treatment. TMS stimulus time is at 0 ms. (**B**): Correlation between increased SalVentAttn-left frontal-insula activity and decreased HAMD-24 score. (**C**): Changes in wPLI phase synchronization in brain network regions of interests in female NSSI patients before and after treatment. (**D**): 77.5 Hz tACS modulates NSSI dynamic emotion-control brain network pattern in female adolescents: 77.5 Hz tACS suppresses activity in the Default network region associated with rumination and self-injurious thoughts, while enhancing the activity of the Limbic, SalVAttn and Control network regions. Concurrently, it strengthens top-down emotional regulation connectivity between the Control over the Default and SalVAttn networks, thereby reducing self-injurious behaviors. Red unidirectional arrows indicate enhanced brain regional activity, blue unidirectional arrows denote reduced activity, red bidirectional arrows represent increased inter-network connectivity, and blue bidirectional arrows signify decreased connectivity. Cont: Control Network; SalVentAttn: Salience Ventral Attention. DLPFC: dorsolateral prefrontal cortex; IPL: inferior parietal lobe; dmPFC: dorsomedial prefrontal cortex; MCC: middle cingulatecortex; OFC: orbitofrontal cortex

TMS-evoked potential (TEP) analysis focused on the C100 (70–150 ms) and C200 (150–260 ms) components to assess the source activity levels and reflect the local brain excitability. After treatment, increased C100/C200 activity was observed in the Limbic (bilateral orbitofrontal cortex, OFC), SalVAttn (bilateral frontal-insula, mid-cingulate), Control (left dorsolateral prefrontal cortex, DLPFC), and Default (left inferior frontal gyrus, frontal operculum) networks (Fig. 2A). Notably, increased C100 activity in the SalVAttn (left frontal-insula) positively correlated with reduced HAMD-24 scores (*R* = 0.826, *p* = 0.043) (Fig. 2B). This correlation analysis was based on a small sample size (*n* = 6), and the correlation coefficient was unstable and highly sensitive to individual data points. The results were only preliminary exploratory findings and needed to be further verified by large-sample studies. Conversely, C200 activity of the Default network (right inferior parietal lobe) decreased (Fig. 2A).

Phase synchronization assessed via wPLI revealed changes in intra- and inter-network connectivity. In the high-gamma (60–90 Hz) band, increased connectivity between the SalVAttn (right frontal-insula) and Default (right precuneus/posterior cingulate) network regions was observed during the C100 component (Fig. 2C, S2A) after treatment. For the C200 component, enhanced connectivity was noted between the Control (left superior parietal region, right mid-cingulate) and SalVAttn (left mid-cingulate) networks, as well as between the Control and Default (right temporal region) networks. Conversely, there was a decrease in connectivity within the Default network (bilateral inferior parietal region), and decreased connectivity between the Default (left inferior parietal region) and SalVAttn (left mid-cingulate) network (Fig. 2C, S2B).

## Discussion

This study demonstrated that 77.5 Hz tACS significantly reduced self-injury behaviors and depressive-anxiety symptoms in female adolescents with NSSI. Furthermore, we observed that at W11, the scores of certain scales showed a slight increase compared to W7, although the scores at W11 still showed a significant decrease compared to the baseline. This observed trend indicates that the symptom improvement effect of 77.5 Hz tACS was sustained to a certain extent during the 8-week follow-up, with only a slight rebound in partial symptoms. After treatment, changes in brain network activity and connectivity were observed, characterized by decreased Default network region activity and intra-network connectivity functional integration. Conversely, increased activity was noted in the Limbic, SalVAttn and Control networks. Additionally, enhanced connectivity was observed between the Control and SalVAttn/Default. These findings may provide novel insights into the neural mechanism underlying the effects of 77.5 Hz tACS on NSSI in female adolescents.

Persistent self-injury persistent thoughts can be viewed as a form of rumination, with self-injury behavior reflecting impaired emotional-control, including the Limbic, SalVAttn, Control and Default networks in NSSI neural mechanisms [4, 5]. The Limbic network centered on the OFC, is responsible for processing emotional information [20] and exhibits reduced activity in NSSI patients [5]. The SalVAttn network anchored in the anterior-insula and cingulate cortex, mediating emotional monitoring, interoception, and pain processing [4, 21], shows diminished SalVAttn-insula regional homogeneity in NSSI patients [22]. The Control network involving the DLPFC and superior parietal lobe and regulating cognitive-emotional control, exhibits functional hypoactivation [18]. Conversely, the Default network including the posterior cingulate/inferior parietal lobe, displays enhanced intra-network activation linked to rumination [23, 24], which correlates with heightened self-injury thoughts [25, 26]. Notably, NSSI patients exhibit weakened connectivity between the Control and SalVAttn networks compared with healthy controls [4, 21, 22]. Previous studies have shown gender differences in the activity of the emotional-control network (e.g., Limbic, SalVAttn networks) in adolescents with NSSI, which may lead to different responses to neuromodulation treatments such as tACS, thus, the findings of this study on network activity changes are only applicable to female adolescents with NSSI.

Our study showed that 77.5 Hz tACS significantly reduced self-injury thoughts and behaviors as well as depressive-anxiety symptoms in female adolescents with NSSI. Utilizing TEPs, we observed that Default-right inferior parietal lobe activity decreased, which may be associated with reduced rumination and thoughts of self-injury [24–26]. Furthermore, Limbic, SalVAttn and Control network regions activity increased, especially in SalVAttn-left insula, which showed a preliminary positive correlation with improvements in depressive symptoms. It is suggested that 77.5 Hz tACS can improve local neuronal activity by entrainment of exogenous high gamma oscillations [27, 28], enhance Salvattn-driven interoceptive awareness and pain sensitivity [29, 30], thereby reducing depressive symptoms and self-injury behaviors (Fig. 2D). However, the C100/C200 changes of TEPs only reflect the changes in local brain excitability and source-level neural activity, and the network-level functional implications need to be verified by combining multiple neuroimaging modalities (e.g., fMRI, MEG) and large-sample studies.

Additionally, exogenous gamma oscillations have been demonstrated to improve neuronal synchronization and synaptic plasticity [11], thereby improving long-range functional connectivity across neural networks [31]. Our results suggested that 77.5 Hz tACS enhanced high-gamma connectivity between regions mediating top-down emotional-control (Control-SalVAttn/Default networks) in female adolescents with NSSI. Therefore, we initially established a brain network dynamic model of 77.5 Hz tACS treatment of NSSI in female adolescents: 77.5 Hz tACS may ameliorate NSSI thoughts by attenuating the hyperactive Default network associated with rumination and self-injury thoughts, while enhancing top-down emotional-control between Control and SalVAttn/Default networks, thereby disrupting negative thought patterns, increasing pain interoception, so as to improving self-injury behavior. This model is a preliminary exploration based on TMS-EEG data of a small sample of female adolescents, and the specific mechanism needs to be further verified by subsequent large-sample and multi-modal studies.

The results of this study share significant similarities with previous research on adult depression using 77.5 Hz tACS, reflecting the same neural physiological mechanisms: Symptom improvement: Whether it is the NSSI of adolescents or adult depression [8, 9, 13], after 77.5 Hz tACS treatment, depressive symptoms (HAMD score) and anxiety symptoms (HAMA score) have significantly decreased, and the effects persist during follow-up. Network regulation:This study suggests that 77.5Hz tACS may reduce default network activity and enhance control-importance network connections, which is consistent with the current research results in depression. Gamma band involvement: Both groups show improvements related to high gamma band synchronization [11, 28], indicating that 77.5 Hz tACS functions as a conservative mechanism across age groups by regulating gamma oscillations. In addition, there are also some differences: Adolescents exhibit stronger neuroplasticity, resulting in faster symptom improvement, and more significant changes in the limbic network activity (orbitofrontal cortex) [5], which may reflect developmental differences in the maturation process of the emotional control network. These differences and similarities need to be further confirmed by comparative studies of different age and gender groups.

Since all the patients included in this study were female, this might be due to the small sample size of the pilot study and the high incidence of self-harm behavior among adolescent females (approximately 70% − 80% of clinical cases in China [3]), but including only female samples would introduce gender bias, as there were differences in neurophysiology between male and female adolescents. The neurophysiological results and conclusions of this study are only applicable to female adolescents with NSSI, and cannot be generalized to male adolescents or the broader NSSI population. Future studies will include males to explore the role of gender as a moderating factor in the neurophysiological mechanisms of NSSI and the therapeutic response to 77.5 Hz tACS.

This study’s findings on high-gamma band connectivity features and TEP changes can be used as interpretable neurophysiological biomarkers for female adolescent NSSI, and combining these biomarkers with interpretable representation learning algorithms can help build an interpretable prediction model for NSSI treatment response, which is conducive to the clinical translation of tACS therapy. In addition, the neurophysiological biomarkers identified in this study need to be improved for cross-domain robustness across genders, age groups and clinical centers by using uncertainty-aware cross-domain adaptation algorithms; future studies should also combine multimodal data (e.g., fMRI, behavioral scales, physiological signals) and adopt advanced multimodal fusion networks to comprehensively analyze the neural mechanism of 77.5 Hz tACS for NSSI and build a more accurate evaluation model of treatment efficacy [32–37]. This study provides preliminary neurophysiological evidence for the application of tACS in adolescent NSSI treatment, and the identified high-gamma band and TEP features can serve as basic biomarkers for subsequent computational modeling and multimodal NSSI recognition research.

This study has four main limitations: first, the small sample size (*n* = 6) may introduce bias and reduce the statistical power of EEG data analysis, with all statistical and correlation results being preliminary exploratory findings that require verification by large-sample studies; second, the absence of sham group limits our ability to exclude placebo effects; third, source activity estimation relied on Brainstorm’s default MNI head model instead of individualized structural MRI models, leading to spatial deviations in source localization, and the reported brain region activations are only regional references rather than precise anatomical localizations; fourth, no structured longitudinal modeling (e.g., growth curve modeling) was used to analyze dynamic symptom changes during follow-up, and the potential factors for slight symptom rebound at W11 need further investigation through longitudinal studies with longer follow-up and more frequent assessments.

Future research should conduct large-sample, double-blind, randomized sham-controlled trials, include healthy control groups for normative comparisons, collect subjects’ individual structural MRI data to use individualized head models for source localization, and combine computational modeling and multimodal data analysis to further validate the therapeutic efficacy of 77.5 Hz tACS for NSSI and clarify its neural network mechanisms.

## Conclusion

77.5 Hz tACS effectively alleviates self-injurious behaviors and depressive-anxiety symptoms in female adolescents with NSSI by regulating functional connectivity, particularly in high gamma frequencies, and enhancing top-down emotional regulation in the emotional-control network. The findings of this study provide preliminary evidence for the application of high-gamma tACS in the treatment of female adolescent NSSI, and the identified neurophysiological features may serve as preliminary biomarkers for subsequent research. However, due to the small sample size and lack of control groups, the results need to be further verified by large-sample, randomized, controlled trials with multi-modal neuroimaging assessments.

## Supplementary Information

Below is the link to the electronic supplementary material.

Supplementary Material 1

## Data Availability

The data supporting the findings of this research were available from the corresponding author upon reasonable request.

## Abbreviations

tACS: Transcranial alternating current stimulation
NSSI: Non-suicidal self-injury
TMS-EEG: Transcranial magnetic stimulation with electroencephalogram
OSIC: Ottawa Self-Injury Inventory Chinese Revised Edition
SalVAttn: Salience Ventral Attention
HAMA: Hamilton Anxiety Scale
HAMD-24: Hamilton Depression Scale-24
PSQI: Pittsburgh Sleep Quality Index
TEPs: TMS-evoked potentials
wPLI: Weighted phase lag index
