# Supplementary material for: High-gamma tACS may regulate brain network connectivity to alleviate symptoms in female adolescent non-suicidal self-injury: a preliminary TMS-EEG pilot study
Source: BMC Psychiatry. 2026 Mar 19;26:346. doi: 10.1186/s12888-026-07978-2 (PMC13122927; doi:10.1186/s12888-026-07978-2)
Supplement: Supplementary file 1 — Supplementary Material 1 [file 12888_2026_7978_MOESM1_ESM.docx]

**eMethods**

**Study Design and Participants**

We recruited six right-handed patients (6 female) diagnosed with adolescent depressive disorder with non-suicidal self-injury behavior at the Department of Neurology of the Hebei Hospital of Xuanwu Hospital, Capital Medical University between December 2024 and January 2025. This study was conducted in accordance with the Declaration of Helsinki and relevant ethical guidelines, with ethics approval [Approval No. LYS (2024) 164] in October 2024 and informed consent obtained from the legal guardians of all patients. This pilot experiment serves as a preliminary exploration for a larger randomized controlled trial (RCT), which has been registered in the Chinese Clinical Trial Registry (ChiCTR) under the identifier ChiCTR2600118169. Inclusion criteria were as follows: (1) meet the DSM-IV diagnostic criteria for depressive disorder; (2) Age 13-17 years old; (3) Patients who did not take antidepressant medications 1 month before recruited or stable antidepressant drugs for more than 4 weeks; (4) HAMD-24 score ≥20; (5) Ottawa Self-Injury Inventory Chinese Revised Edition (OSIC) score: the frequency of self-injury thought and behavior in the past 6 months is not less than 2 points [1]; (6) The legal guardian signed the informed consent. Exclusion criteria were as follows: (1) Patients with severe physical or brain diseases; (2) Patients with a history of attempted suicide within 1 year; (3) HAMD-24 item-3 (suicide-item)≥3; (4) Received electroconvulsive therapy, transcranial magnetic stimulation therapy and other physical therapy within 1 month; (5) Changes in drug dose during treatment; (6) Patients with a history of drug, alcohol or other psychoactive substance abuse; (7) Patients with pacemakers, coronary stents and aneurysm clips.

**The neuropsychological scale assessment**

Neuropsychological scale assessments were performed on all patients with NSSI by doctors specializing in psychosomatic diseases who did not know the treatment tasks. All subjects were evaluated four times at baseline, at the end of treatment (W3), at the 4-week follow up (W7) and at the 8-week follow up (W11). The neuropsychological scale included Ottawa Self-Injury Inventory Chinese Revised Edition (OSIC), Hamilton Depression Scale-24(HAMD-24), Hamilton Anxiety Scale (HAMA)and Pittsburgh Sleep Quality Index Score (PSQI). The occurrence and severity of adverse events were assessed using the Treatment Emergent Symptom Scale and Adverse Event Scale. The primary outcome was OSIC score, and the secondary outcome included HAMD-24 score, HAMA, PSQI score and adverse events.

**Data acquisition and preprocessing of TMS-EEG**

TMS stimulation was performed using the Magstim Transcranial magnetic Stimulation Instrument (Magstim Company Ltd., London, UK), with a maximum stimulus output intensity of 2.0 Tesla. TMS-EEG data were obtained using a magnetic field-compatible EEG amplifier with a sampling rate of 1,024 Hz (Yunshen Ltd, Beijing, China) and an electrode cap with 128 TMS-compatible electrodes according to a 10-20 system (Greentek Ltd, Wuhan, China). The electrode impedance was maintained below 5 kΩ. Electrodes CPz and nose tip were used as ground and reference electrodes, respectively. A total of 150 stimulus of sTMS was administered to the left frontal lobe (corresponding to F3 points on the subject’s scalp, according to the international 10–20 system) through a figure-eight coil with a diameter of 70mm. The stimulation intensity was 90% of the (Rest motor threshold, RMT). Each stimulus was provisioned at an interval of 4s, lasting for 8 minutes. RMT was defined as the minimum stimulation intensity capable of evoking MEP amplitude 50 μ v at 5 out of 10 stimuli[2].


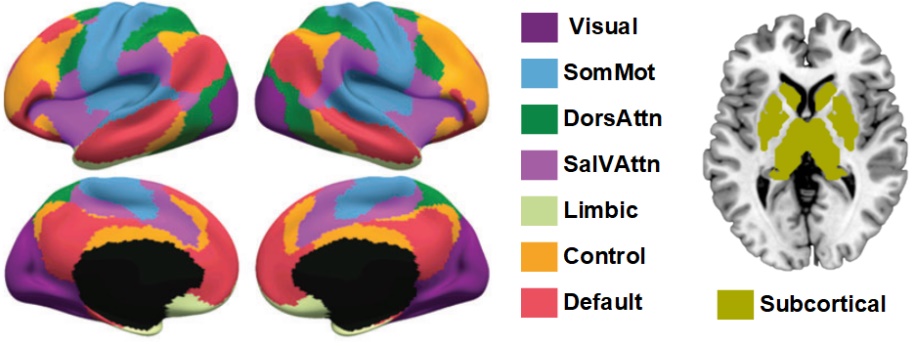


### **Fig S1.** Whole-brain functional parcellation.

We performed the Hilbert transform on the TMS-EEG data and then analyzed the wPLI in the high gamma(60-90Hz) band. Studies have shown that wPLI is very sensitive in describing the synchronization of EEG time series. Therefore, we used wPLI to quantify the strength of functional connections between nodes and construct functional brain networks. The specific calculation formula of wPLI was as follows[3]:


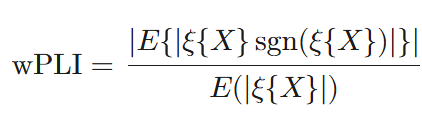


Where X refers to the spectral cross spectrum of two time series, and ξ represents the virtual part of the cross spectrum. wPLI values are typically between 0 and 1, where 0 indicates no synchronization between the two time series and 1 indicates complete synchronization.

We constructed a weighted undirected network by defining 100 ROIs as network nodes and wPLI values between brain regions as edges. The upper triangular part of the wPLI matrix was extracted to construct one feature vector for each subject.To improve reliability, we averaged the calculated individual connected networks to calculate the representative connected networks.

**Statistics**

**Demographic and clinical efficacy analysis**

Statistical analysis was performed using SPSS 26 (SPSS Inc., Chicago, IL) and GraphPad Prism （V8.0）. The Shapiro-Wilk test was used to evaluate the normality of continuous data. All clinical scale data were consistent with the normal distribution, describing as mean ± error. The analysis of OSIC, HAMD-24, HAMA and PSQI scores at four time points (W0, W3, W7 and W11) was conducted using mixed repeated measures ANOVA. Mauchly sphericity test was used to evaluate whether the data satisfied the spherical distribution. If not, Greenhouse-Geisser correction was used, followed by post-hoc analysis and Bonferroni correction. Pairwise comparisons were conducted three times for within-groups (W3/W7/W11 vs. W0, P <0.017 (0.05/3)).

**TEPs and wPLI data analysis**

Both TEPs and wPLI data were statistically analyzed using matlab, GraphPad Prism (V8. 0, GraphPad Software) and SPSS 26 (SPSS Inc., Chicago, IL). The R package pheatmap (version 1.0. 8, The R Project for Statistical Computing) was used to generate heatmaps.

By comparing the average butterfly plots of the TEPs of six NSSI patients before and after treatments in this trial and considering that early components were susceptible to TMS stimulation, we focused on the two most reliable TEPs components:C100(70-150ms) and C200(150-260ms). TEPs amplitudes were averaged over this time window.

If the quantitative data conformed to the normal distribution, the data were described as mean ± standard error, using a paired t-test to compare the average TEPs and wPLI data before and treatments in NSSI patients. P < 0.05 was considered to be statistically different. The pre - and post-treatment differences in TEPs were mapped onto a brain topology.

**References**

1. Zhu J, Qian R, Zhong H, Li Y, Liu X, Ma J. Factors influencing the addiction characteristics of non-suicidal self-injurious behaviors in adolescents: A case-control study. Front Psychiatry. 2022;13:1033242. https://doi: 10.3389/fpsyt.2022.1033242.
2. Rossini PM, Burke D, Chen R, Cohen LG, Daskalakis Z, Di Iorio R, et al. Non-invasive electrical and magnetic stimulation of the brain, spinal cord, roots and peripheral nerves: Basic principles and procedures for routine clinical and research application. An updated report from an I.F.C.N. Committee. Clin Neurophysiol. 2015; 126(6):1071-1107. https://doi: 10.1016/j.clinph.2015.02.001.
3. Ma J, Wang Z, Cheng T, Hu Y, Qin X, Wang W, et al. A prediction model integrating synchronization biomarkers and clinical features to identify responders to vagus nerve stimulation among pediatric patients with drug-resistant epilepsy. CNS Neurosci Ther. 2022;28(11):1838-1848. https://doi: 10.1111/cns.13923.

**Table S1 Neuropsychological scale in NSSI patients at four times (W0, W3 , W7 and W11)**

| Scales |  | Times (mean ± SE) | | | | F | P | Cohen's d |
| --- | --- | --- | --- | --- | --- | --- | --- | --- |
|  |  | W0 | W3 | W7 | W11 |  |  |  |
| OSIC*(n=6)* |  | 57.33±9.66 | 32.17±8.77 | 18.50±7.11* | 27.50±9.83 | 5.677 | 0.008 | \| -1.335^a^, -1.682^b^, -1.038^c^ \| \| --- \| |
|  | Thought and Behavior Frequency | 13.67±2.17 | 7.83±1.85 | 3.00±1.61* | 5.50±1.52 | 9.894 | 0.001 | -1.299^a^,-2.114^b^,-1.352^c^ |
|  | Social Influence | 10.17±2.41 | 4.17±2.09 | 3.17±1.08 | 5.33±2.17 | 3.152 | 0.056 | -1.081^a^,-1.295^b^,-0.741^c^ |
|  | External Emotion Regulation | 9.17±1.99 | 6.17±1.89 | 3.50±1.46 | 6.00±2.15 | 2.470 | 0.102 | -0.584^a^,-0.985^b^,-0.497^c^ |
|  | Internal Emotion Regulation | 10.00±2.35 | 5.00±1.97 | 3.67±1.82 | 4.00±1.81 | 3.900 | 0.030 | -1.443^a^,-1.208^b^,-0.909^c^ |
|  | Addiction | 14.33±2.12 | 9.00±2.77 | 5.17±1.76* | 6.67±2.82 | 3.624 | 0.038 | -0.928^a^,-1.820^b^,-1.090^c^ |
| HAMD-24  *(n=6)* |  | 33.00±5.15 | 21.67±5.51* | 7.50±2.59* | 6.83±2.71* | 26.585 | 0.000 | -1.892^a^,-2.839^b^,-2.738^c^ |
| HAMA*(n=6)* |  | 25.33±3.50 | 15.33±3.31 | 10.00±1.46* | 8.50±3.47* | 18.810 | 0.000 | -1.327^a^,-2.081^b^,-2.467^c^ |
| PSQI*(n=6)* |  | 12.67±1.50 | 7.83±1.60* | 4.33±0.84* | 6.00±0.73* | 16.606 | 0.000 | -1.516^a^,-3.858^b^,-1.969^c^ |

NSSI:Non-suicidal self-injury; OSIC, Ottawa Self-Injury Inventory Chinese Revised Edition; HAMD-24, Hamilton Depression Scale-24; HAMA, Hamilton Anxiety Scale; PSQI, Pittsburgh Sleep Quality Index. ^*^P<0.017 vs. W0; W0: at baseline; W3: after treatments at week 3; W7: 4-week follow-up at week 7. W11: 8-week follow-up at week 11; a:W3 vs W0; b:W7 vs W0; c:W11 vs W0.


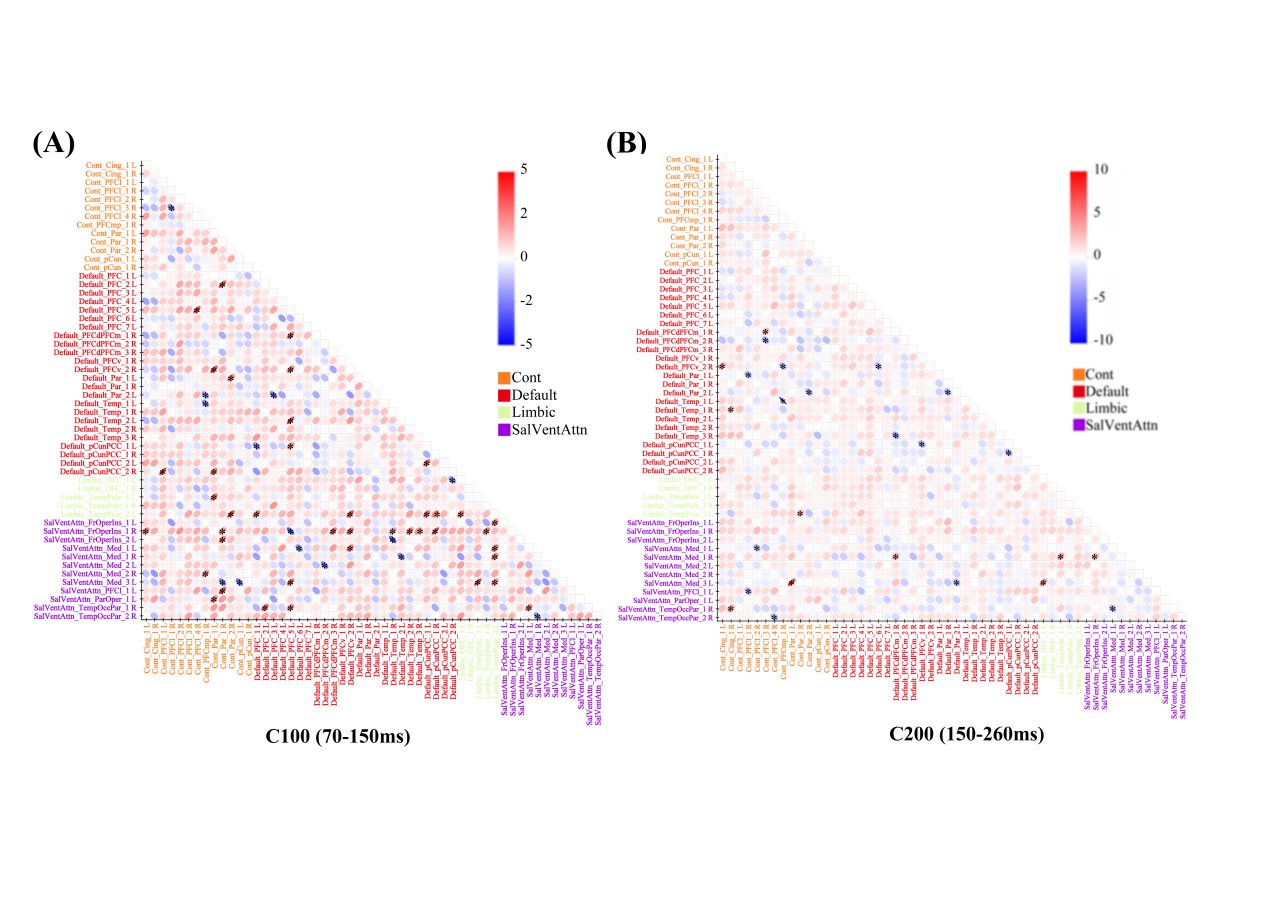


**Fig S2.** Changes of wPLI phase synchronization in brain network all cortical regions in NSSI patients before and after treatment. Cont: Control Network； DorsAttn: Dorsal Attention Network；SalVentAttn: Salience Ventral Attention. ^*^P<0.05.
